# Microcystic lymphatic malformation following a double mastectomy

**DOI:** 10.1016/j.jdcr.2023.12.011

**Published:** 2024-01-01

**Authors:** Dina Poplausky, Austin J. Piontkowski, Robert G. Phelps, Nicholas Gulati

**Affiliations:** Department of Dermatology, Icahn School of Medicine at Mount Sinai, New York, New York

**Keywords:** acquired lymphatic malformation, lymphangioma circumscriptum, microcystic lymphatic malformation

## Introduction

Abnormalities of the lymphatic system can result in lymphatic malformation, a broad term referring to a class of conditions. One such condition is microcystic lymphatic malformation (MLM), also known as lymphangioma circumscriptum, which is an uncommon dilation of the lymphatic channels. It is superficial and lesions measure <1 centimeter in longest diameter. MLM presents as grouped, translucent, or hemorrhagic vesicles with a “frogspawn” appearance.^1^ Although a benign entity, it carries risks of complications including infection and bleeding.^2^^,^^3^ MLM can be congenital or acquired due to alterations or blockages of the lymphatic system.^1^ The acquired type is typically seen following surgery and/or radiation. It is a late complication, occurring several years after oncologic treatment.^4^, ^5^, ^6^ To the authors’ knowledge, this is the first reported case of MLM after mastectomy only, without radiation.

## Case history

A 59-year-old female presented to dermatology with a reported ten-year history of abdominal blisters, starting several months after a double mastectomy for breast cancer. She had never received chemotherapy or radiation. The blisters were asymptomatic, but occasionally drained blood. She had been prescribed multiple topical steroids and antifungals empirically without any improvement. She had no history of autoimmune diseases. Her history was negative for any temporal associations with any topical or oral medication.

**Fig 1:**
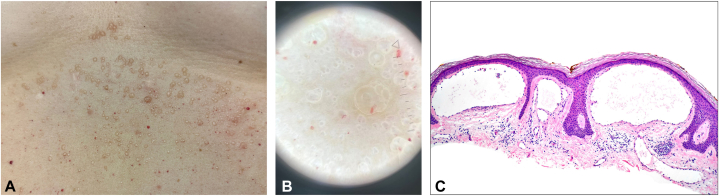
**A,** Grouped vesicles primarily on the central abdomen. **B,** Dermoscopy demonstrating *yellow* lacunae surrounded by pale septae and vascular structures. **C,** Hematoxylin and eosin stain of a skin biopsy at 20× magnification demonstrating ectatic lymphatic vessels filled with proteinaceous fluid impinging on and enclosed by the lower epidermis.

On physical examination, there were small, translucent vesicles primarily on the central abdomen (Fig 1, *A*). The vesicles did not rupture with light or firm palpation. The dermoscopic evaluation revealed yellow lacunae surrounded by pale septae and vascular structures (Fig 1, *B*). A skin biopsy demonstrated cystically dilated lymph vessels lined by simple endothelium containing proteinaceous fluid in the superficial dermis, with the dilated vessels impinging on and contained by the epidermis. These findings are consistent with MLM (Fig 1, *C*).

**Fig 2:**
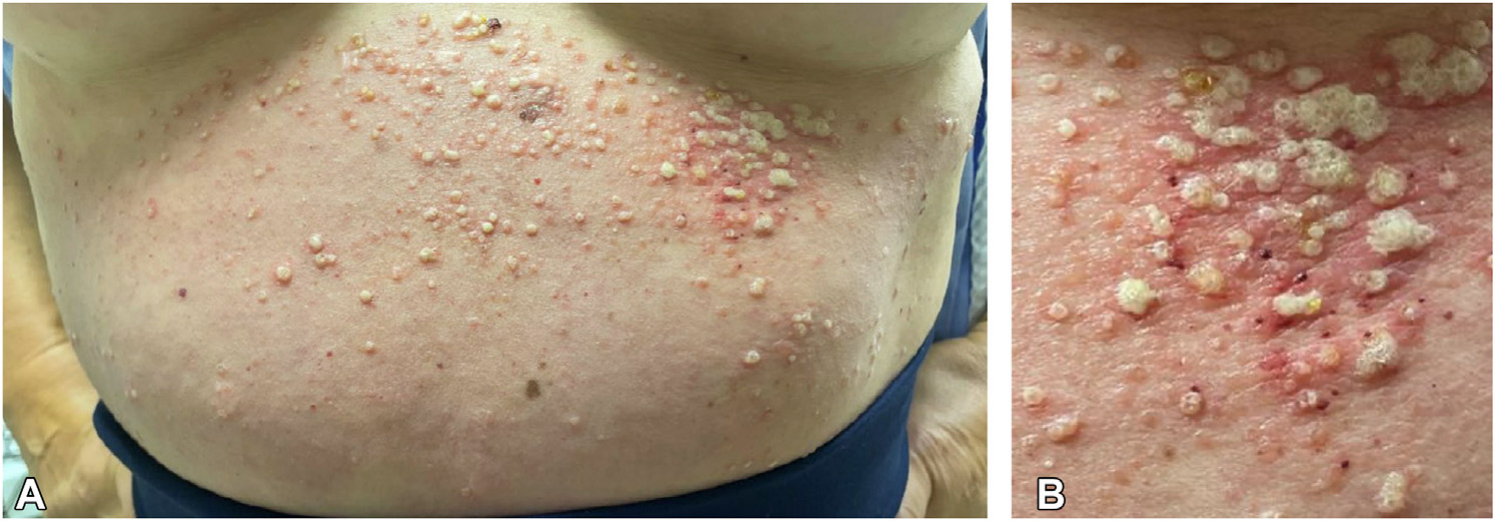
**A, B,** Grouped pustules with underlying erythema on the patient’s central abdomen six weeks after initial presentation.

Six weeks later, she presented to the emergency department with a fever and changes in her skin findings. Her exam revealed grouped pustules with underlying/background erythema at the same locations as her prior translucent vesicles (Fig 2). She was diagnosed with bacterial cellulitis as a complication of MLM, a complication the patient notes having suffered from four years earlier. After five days of treatment with cephalexin 500 mg four times daily and trimethoprim-sulfamethoxazole 800-160 mg twice daily, the patient became afebrile and returned to her baseline skin findings.

## Discussion

The literature on acquired MLM in adulthood remains sparse. Because the condition is not fully understood, prolonged misdiagnosis of MLM in an adult patient presenting with vesicular lesions is not uncommon. The differential diagnosis of MLM includes miliaria crystallina, venous malformations, cutaneous metastases, contact dermatitis, molluscum contagiosum, and herpes simplex virus infection.^1^ In the setting of surgically treated breast cancer, particularly with adjuvant radiotherapy, it is essential to equally consider lymphangiomatous atypical vascular lesions and angiosarcoma.^7^^,^^8^ Due to the similarities in clinical morphology, MLM should be considered in cases of persistent vesicular papules. Histopathologic evaluation of a suspected lesion showing cystically dilated lymph vessels is suggestive of MLM.

Management of MLM is difficult and is often centered around symptomatic treatment, including topical steroids for itch. Surgical excision is typically the primary treatment modality, but it may not be feasible given the extent of disease for some patients, as in this case, and medical history contraindications. Additionally, laser procedures are emerging as a promising option, not only for therapeutic purposes, but also for cosmetic enhancement.^9^

This case presents a unique and noteworthy instance of MLM manifesting as grouped vesicles in a patient with a history of breast cancer and subsequent mastectomy. To our knowledge, this is the first reported case of MLM that occurred in the setting of mastectomy alone, and one of the few documented cases of acquired MLM in adulthood. However, it is important to note that this is a single case, and the lesional distribution across the upper abdomen leaves open the possibility that these findings are unrelated to the patient’s surgical history. The prolonged duration of the condition, coupled with the patient's lack of response to empirically prescribed topical steroids and antifungals, underscores the diagnostic challenges associated with MLM. The emergence of cellulitis as a complication further emphasizes the importance of timely diagnosis and intervention of this condition. Recognizing MLM at an early stage is pivotal, as it facilitates timely referrals for treatment, reduces the risk of complications, and ultimately leads to more favorable cosmetic outcomes for patients.

## Conflicts of interest

None disclosed.
